# The UK Longitudinal Linkage Collaboration: A trusted research environment for the longitudinal research community

**DOI:** 10.23889/ijpds.v8i2.2299

**Published:** 2023-09-14

**Authors:** Andy Boyd, Robin Flaig, Jacqui Oakley, Kirsteen Campbell, Katharine Evans, Stela McLachlan, Richard Thomas, Emma Turner

**Affiliations:** 1 University of Bristol, Bristol; 2 University of Edinburgh, Edinburgh

## Objectives

Our Trusted Research Environment (TRE) provides a centralised infrastructure to pool Longitudinal Population Studies’ (LPS) data and systematically link participants’ routine health, administrative and environmental records. All data are held in a centralised research resource which is now certified by UK Statistics Authority as meeting the Digital Economy Act standard.

## Methods

We have created an unprecedented infrastructure integrating data from interdisciplinary and pan-UK LPS linked to participants’ NHS England records with delegated access responsibilities. Integrated and curated data are made available for pooled analysis within a functionally anonymous DEA and ISO 27001 accredited TRE. We developed a bespoke governance and data curation framework with LPS data managers and Public/participant contributors. New data pipelines are being built with partners at ADRUK and the Office of National Statistics to link non-health records. Our design supports long-term sustainability, linkage accuracy and the ability to link data at both an individual and household level.

## Results

This organisation is a collaboration of >24 LPS with ~280,000 participants. Participants' data are linked to NHS records and geo-coded environmental exposures. This resource is now accessible for public benefit research for bona fide UK researchers. Administrative data including tax, work and pensions, and education are being added to the resource. This data flow is enabled by: (1) a model where TTP processes participant identifiers for many different data owners; (2) creation of a novel longitudinal data pipeline, enabling linkage, data extraction and update of records over time; (3) an access framework where Linked Data Access Panel considers applications on behalf of data owners (e.g., the NHS), with review by a Public Panel and distributing applications to LPS for approval of appropriate data use.

## Conclusion

Our organisation provides a strategic research-ready platform for longitudinal research. We are extending linkages of LPS participants to previously inaccessible datasets. The research resource is positioned to allow researchers to investigate cross-cutting themes such as understanding health and social inequalities, health-social-environmental interactions, and managing the COVID-19 recovery.

